# GWAS-Assisted Genomic Prediction to Predict Resistance to Septoria Tritici Blotch in Nordic Winter Wheat at Seedling Stage

**DOI:** 10.3389/fgene.2019.01224

**Published:** 2019-11-26

**Authors:** Firuz Odilbekov, Rita Armoniené, Alexander Koc, Jan Svensson, Aakash Chawade

**Affiliations:** ^1^Department of Plant Breeding, Swedish University of Agricultural Sciences, Alnarp, Sweden; ^2^Institute of Agriculture, Lithuanian Research Centre for Agriculture and Forestry (LAMMC), Akademija, Lithuania; ^3^Nordic Genetic Resource Centre, Alnarp, Sweden

**Keywords:** GWAS - genome-wide association study, genomic prediction (GP), genomic selection (GS), wheat, Septoria tritici blotch (STB), Quantitative trait loci (QTL)

## Abstract

Septoria tritici blotch (STB) disease caused by *Zymoseptoria tritici* is one of the most damaging diseases of wheat causing significant yield losses worldwide. Identification and employment of resistant germplasm is the most cost-effective method to control STB. In this study, we characterized seedling stage resistance to STB in 175 winter wheat landraces and old cultivars of Nordic origin. The study revealed significant (*p* < 0.05) phenotypic differences in STB severity in the germplasm. Genome-wide association analysis (GWAS) using five different algorithms identified ten significant markers on five chromosomes. Six markers were localized within a region of 2 cM that contained seven candidate genes on chromosome 1B. Genomic prediction (GP) analysis resulted in a model with an accuracy of 0.47. To further improve the prediction efficiency, significant markers identified by GWAS were included as fixed effects in the GP model. Depending on the number of fixed effect markers, the prediction accuracy improved from 0.47 (without fixed effects) to 0.62 (all non-redundant GWAS markers as fixed effects), respectively. The resistant genotypes and single-nucleotide polymorphism (SNP) markers identified in the present study will serve as a valuable resource for future breeding for STB resistance in wheat. The results also highlight the benefits of integrating GWAS with GP to further improve the accuracy of GP.

## Introduction

Septoria tritici blotch (STB) disease caused by fungal pathogen *Zymoseptoria tritici* is one of the devastating foliar diseases of wheat in the temperate regions worldwide. STB causes significant yield losses and additional fungicide expenses (Fones and Gurr, 2015; Torriani et al., 2015). The annual harvest losses reach 5% to 10% in the biggest EU wheat producing countries (Fones and Gurr, 2015). Cultivation of resistant cultivars in combination with fungicide application is the main strategy to control the disease. Besides, a major problem of the intensive use of fungicides is that many populations of *Z. tritici* have rapidly evolved resistance to its active agents (Torriani et al., 2009; Wieczorek et al., 2015; Cheval et al., 2017). Therefore, novel sources of resistance to STB and their introgression into wheat breeding programs is the most economical and environmentally friendly strategy for effective management of the disease.

So far, 21 genes are mapped for resistance to STB in wheat (Brown et al., 2015). The expression pattern and effect of these genes on resistance to STB differ in seedling and adult plant stages. For example, *Stb16* is expressed and effective at the seedling and adult stages of plants while *Stb17* is expressed only at the adult stage (Tabib Ghaffary et al., 2011). *Stb18* is an isolate-specific resistance gene that shows variable resistance to *Z. tritici* at seedling and adult stages depending on the isolate (Tabib Ghaffary et al., 2011). *Stb6* and *Stb15* are the two most common STB resistance genes in the current European germplasm (Arraiano and Brown, 2006). *Stb15* was found in about 60% of cultivars tested but, unlike *Stb6, Stb15* is not known to show resistance under field conditions (Arraiano et al., 2009; Brown et al., 2015). The only qualitative gene for STB resistance *Stb6* (Saintenac et al., 2018) and recently identified avirulence gene *AvrStb6* of *Z. tritici* (Zhong et al., 2017) have been shown to be in a gene-for-gene relationship. *Stb6* is among the most frequent STB genes in European wheat germplasm and suggested as the most widespread STB gene in the contemporary wheat breeding programs (Arraiano and Brown, 2017). However, this gene alone is not sufficient to provide adequate resistance to STB, and there are no other known resistance genes contributing significantly to the reduction of *Z. tritici* populations in Europe (Arraiano et al., 2009). The majority of variation in field resistance to STB is controlled by quantitative resistance, and the progress in breeding for STB resistance over the last 30 years presumably happened by the gradual accumulation of minor genes. Recently, it was shown that the STB disease symptoms chlorosis, necrosis, and pycnidia are under varying genetic control (Odilbekov et al., 2019). Therefore, there is a need to search for new sources of durable disease resistance to STB for marker-assisted introgression into elite wheat cultivars (Fones and Gurr, 2015; McDonald and Mundt, 2016).

Wheat landraces are a valuable source of genetic diversity. They are adapted to the environmental conditions of their place of origin and thus can provide novel sources of disease resistance for developing new cultivars adapted to the changing climate (de Carvalho et al., 2012; Lopes et al., 2015). Several useful agronomic and resistance traits have been introgressed from landraces to commercial wheat cultivars including the dwarfing gene *Rht* from the Japanese landrace Shiro Daruma (Dreisigacker et al., 2005) and the high grain protein content gene *NAM-B1* in Fennoscandian wheat (Hagenblad et al., 2012). Valuable landraces and old cultivars of winter wheat consisting of more than 300 genotypes from Scandinavian countries is preserved at the Nordic Genetic Resource Centre (NordGen, Alnarp, Sweden), and part of this material was evaluated earlier for several agronomic traits and showed high diversity in morphological traits (Diederichsen et al., 2012), resistance to rust (Randhawa et al., 2016) and powdery mildew (Hysing et al., 2007). These studies prove that the material stored at NordGen is unique and a genetically diverse resource, which can be utilized for the improvement of wheat cultivars for Nordic and Baltic Sea Region countries (Chawade et al., 2018).

Genome-wide association studies (GWAS) and genomic selection (GS), both performed with genome-wide markers are important and effective tools for plant breeding. GWAS estimates marker effects across the whole genome on the target population based on prediction models (Desta and Ortiz, 2014). Based on linkage disequilibrium (LD), GWAS can identify new functional alleles (identify novel genes and QTLs) for many agriculturally important traits in diverse germplasm. Few GWAS studies were performed for STB resistance in European winter wheat accessions (Kollers et al., 2013; Miedaner et al., 2013; Vagndorf et al., 2017). Many regions associated with resistance to STB in the wheat genome were identified in these studies. In a study of 1,055 elite hybrids and their corresponding 87 parental lines, Miedaner et al. (2013) identified four significant single-nucleotide polymorphisms (SNP) associated with STB resistance located on chromosomes 1B, 2B, 5B, and 6A. Kollers et al. (2013) detected 39 SSR on 2A, 2D, 3A, 5B, 7A, 7D significantly associated with adult plant resistance in a panel of 372 European wheat lines. Four QTL, on chromosomes 1B, 2A, 5D, and 7A were highly associated with STB resistance in 164 North European cultivars and breeding lines (Vagndorf et al., 2017).

GS, on the other hand, enables the selection of superior genotypes based on genomic estimated breeding values (GEBV) to create models for the prediction of phenotypes in uncharacterized populations (Meuwissen et al., 2001). Previous studies have shown the feasibility of GS for predicting STB resistance in wheat. Juliana et al. (2017) achieved a mean genomic prediction (GP) accuracy of 0.45 for adult plant resistance to STB in a population of 333 and 314 advanced lines from Centro Internacional de Mejoramiento de Maíz y Trigo‘s (CIMMYT) wheat breeding program. Muqaddasi et al. (2019) investigated the potential of GP of adult stage STB infection in a European winter wheat panel of 371 elite varieties, resulting in both additive and non-additive prediction models centered around a mean GP accuracy of approximately 0.43. Spindel et al. (2016) described the new combined GS + GWAS model based only on the results of GWAS run using GS training population data. GS + GWAS has some benefits as the method does not require additional data as the same phenotype and genotype data set is used, prediction accuracy can be enhanced, and it can be more accessible to breeders as it does not require extensive knowledge on the underlying genetics of a trait of interest (Spindel et al., 2016).

Previous studies were primarily focused on resistance to STB in the adult stage of winter wheat germplasm. One of the main goals of this project was to characterize seedling stage resistance to STB in winter wheat landraces and old cultivars of Nordic origin which are well adapted to the Nordic climate. The current study relies on a collection of 175 winter wheat accessions, released between 1900 and 2012. In this work, this material was evaluated for seedling-stage resistance to STB disease. The objectives of this study were (i) to detect novel STB disease resistance loci at the seedling stage by performing GWAS analysis; (ii) to identify candidate genes to STB resistance in wheat; (iii) to evaluate GP (GP) for selection for STB resistance; and (iv) to employ GP+GWAS to further improve the accuracy of GP.

## Materials and Methods

### Plant Material

The material in this study comprised of 175 winter wheat cultivars and landraces (hereafter genotypes) mainly from Scandinavian countries (**Supplementary Table 1**). The collected genotypes were released between 1900 and 2012 and representing a century of winter wheat breeding history of the region. Four genotypes originating from Germany were also included as they have been widely grown in the Scandinavian area. The seeds were obtained from Nordic Genetic Resources Centre, Alnarp, Sweden (NordGen).

### Growth Conditions

The seeds were placed on a moist filter paper in Petri dishes and kept for 4 days at +4°C in dark. Afterwards, they were transferred to room temperature conditions for two days for germination. Thereafter, the germinated seeds were sown in plastic pots (8 × 8 × 8 cm) filled with peat substrate. Two seeds of each genotype were sown per pot. Plants were grown in the Biotron greenhouse chamber at 24°C with a 16-h photoperiod and 60% humidity. The light intensity was set and controlled at 250 µmol m^−2^·s^−1^. The samples were arranged in an augmented design with eight blocks designed with the R package Agricolae (Mendiburu, 2017). Four genotypes were used as checks in each block to control block effect, namely, Nimbus (susceptible), Nelson and Target (moderately resistant), and Kranich (resistant). The entire experiment was performed twice with 1-month interval, and two replications were done at each occasion.

### Inoculation and Disease Assessment

The *Z. tritici* strain was isolated from typical STB lesions on leaves of winter wheat collected in southern part of Sweden during 2015, and the inoculum was prepared as described previously (Odilbekov et al., 2018). Second and third leaves of the seedlings were marked close to the stem with a permanent marker before inoculation. The twentyone day old wheat seedlings were inoculated with *Z. tritici* inoculum using a hand sprayer with a spore concentration of 10^7^ spores ml^–1^. The inoculum was applied on the leaves three times, and leaves were allowed to dry for 1 h each time. The inoculated seedlings were transferred to fully controlled daylight chamber and kept 72 h under close to 100% relative humidity at 24°C with a 16-h photoperiod and a light intensity of 250 µmol m^−2^·s^−1^. Relative humidity was reduced to 65% 72 h post-inoculation. Percentage of the necrotic area on the inoculated leaf surface (from 0% to 100%) was visually scored at 13, 16, and 19 days post-inoculation (dpi). The lesion development over the assessment period was summarized through the computation of the relative area under the disease progress curve (rAUDPC). The entire experiment was repeated twice.

### Genotypic Data and Population Structure

The samples for DNA extraction were collected from 6-week-old seedling and the DNA extraction and genotyping of the samples was performed by TraitGenetics GmbH, Germany (www.traitgenetics.com/en/). The samples were genotyped with a 20K SNP wheat marker array. A total of 6,097 SNPs were used for GWAS after removing SNPs with more than 20% missing data as well as a minor allele frequency less than 5%. Principal component analysis (PCA) was done with the software Simca 14 (Umetrics, Sweden).

### GWAS and GP

GWAS analysis was done with the GAPIT package (v3.0) in R (Tang et al., 2016). The primary model was constructed with the GLM algorithm (Lipka et al., 2012) with 10 principal components as covariates and MAF threshold of 0.05. A QTL was considered significant at the threshold of adjusted false discovery rate (FDR) < 0.05. New GWAS models were developed using MLM, MLMM, FarmCPU, and Super algorithm in GAPIT for verification of the QTL obtained with GLM. GP modeling was done using the R package rrBLUP (v4.6) (Endelman, 2011) for ridge-regression-based genome-wide regression. The rrBLUP model for genome-wide regression assumes the form *y = Xb + Zu*, where *X* and *Z* are the design matrices for fixed and random effects, respectively, *b* and *u* are vectors of fixed and random effects, and *y* is a vector of phenotypic values. Similar to the method proposed by Spindel et al. (2016), significant markers identified by GWAS results were included as fixed effects in the GS model and removed from the design matrix of random effects. To identify the best subset of GWAS-selected markers to include as fixed effects, all possible permutations of available GWAS-selected markers, were evaluated with respect to average model accuracy. Number of markers in the marker sets ranged from one (a single marker added as fixed effect) to five (all available markers). The GP models were validated on a set of 500 random 80/20 train/test set splits. Model accuracy was assessed by calculating Pearson’s correlation coefficient between the predicted and observed STB resistance for each of the train/test sets and estimating the average of all correlation values for each run. The best performing model was selected on the basis of the highest average model accuracy. The GP models with markers fitted as fixed effects were compared to a GP model which did not use GWAS-selected markers as fixed effects, instead fitting all available markers as random effects, and was also compared to models that mimicked the model configuration of the fixed effect models described above, but which instead sampled random markers (as opposed to selecting markers based on highest significance in a GWAS). The subset sizes used for the models using the randomly selected markers ranged from one to five. Each subset size was evaluated five times, with a new random draw of markers. The initially described model which fit all markers as random effects, and the models fitting randomly selected markers as fixed effects, were all validated against the same 500 train/test splits as the GWAS-selected marker models.

### Identification of Candidate Genes

The physical positions of the significant markers from the GLM model were identified by BLASTing their sequences against the IWGSC RefSeq v1.0 genome. The physical location of flanking markers BobWhite_c42716_71 and BS00110231_51 fell into range of 623,712,765 to 623,989,423 bp in the region of chromosome 1B. The candidate genes physically located within this range were identified, and their gene annotation was extracted from IWGSC RefSeq v1.0 genome.

## Results

### Phenotypic Diversity

**Figure 1:**
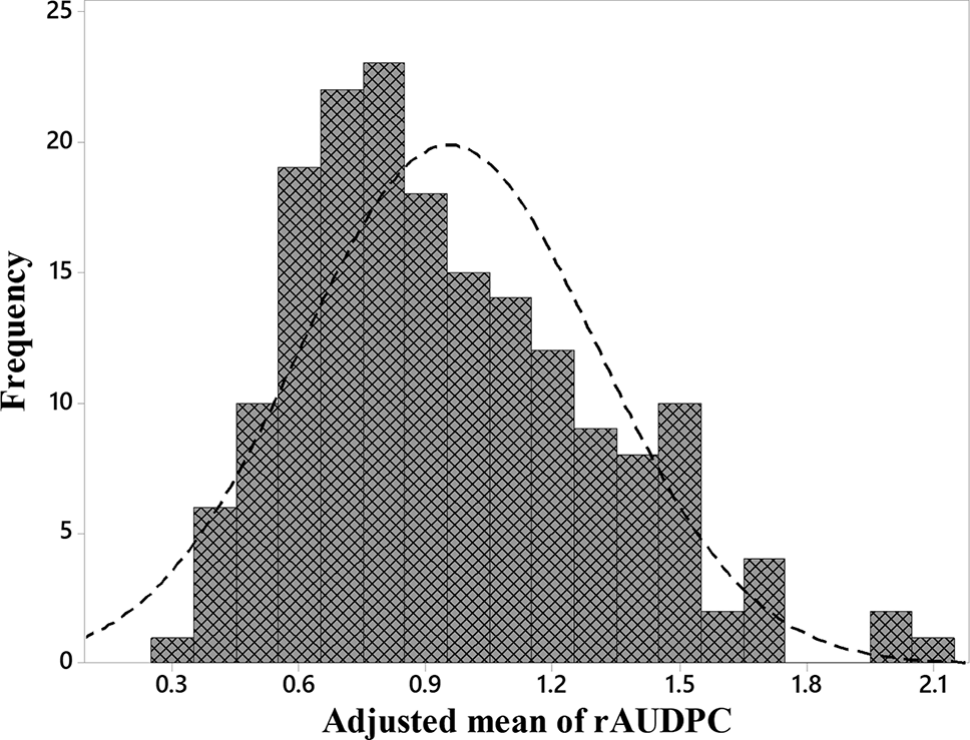
Frequency distribution of adjusted rAUDPC mean of STB score of two greenhouse experiments.

The *Z. tritici* isolate was evaluated on a differential set of wheat cultivars with known *Stb* resistance genes (**Supplementary Figure 1**). The evaluation of 175 genotypes showed that the phenotypic distribution of STB severity followed approximately a normal distribution (**Figure 1**). Highly significant (*p* < 0.05) phenotypic differences in STB severity were observed in the germplasm (**Table S1**). The mean of the rAUDPC values ranged from 0.33 for the most resistant and 2.07 for most susceptible genotypes, respectively. Tukey multiple comparison test showed that the genotypes Kranich, Starke, Galicia, and Cymbal exhibited a higher level of resistance to STB while the lower level of resistance was found in genotypes such as Penta, Sejet, Svea I, and Gluten.

### Population Structure

**Figure 2:**
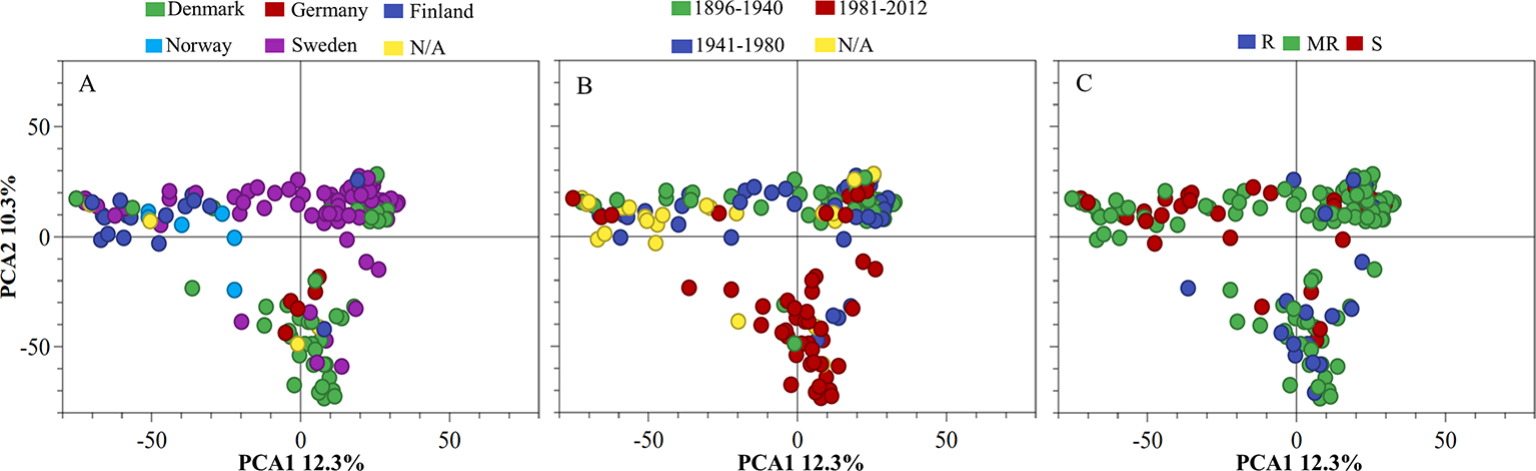
Principal component analysis (PCA) of 175 winter wheat cultivars/landraces coloured and labelled by **(A)** country of origin, **(B)** released year and **(C)** resistance/susceptibility. PCA was based on the allele frequencies of 6097 SNP markers. R, resistant; MR, moderate resistant; S, susceptible.

**Figure 3:**
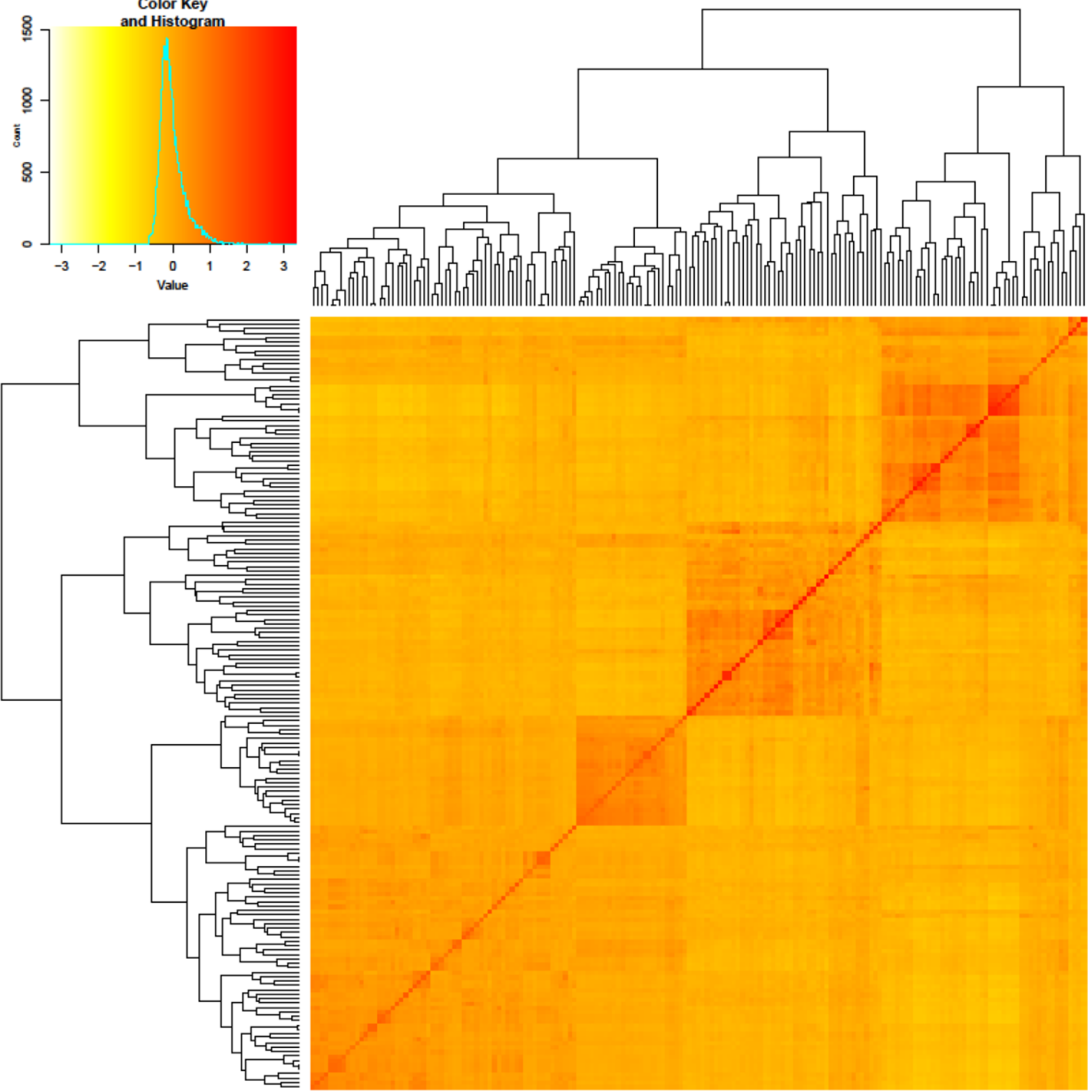
Heatmap and dendrogram of a kinship matrix among 175 winter wheat cultivars/landraces estimated using the SNP data.

To identify underlying genetic differences, PCA and Kinship analyses were performed on the genotypes based on 6,097 SNPs. The first and second principal components accounted for 12.3% and 10.03% of the variance, respectively. The genotypes were clustered into three major groups, and the clustering was mainly based on their geographic origin (**Figure 2A**). The genotypes with origin from Denmark and Finland formed two very distinct clusters, whereas the Swedish genotypes could be considered intermediate between these two clusters. The result from PCA revealed that most of the genotypes with a higher level of resistance belong to the modern wheat cultivars while most of the susceptible genotypes belonged to older released ones (**Figures 2B, C**). A similar result to PCA was also observed by using Kinship analysis where three different clusters were identified (**Figure 3**).

### Genome-Wide Association Analysis

**Figure 4:**
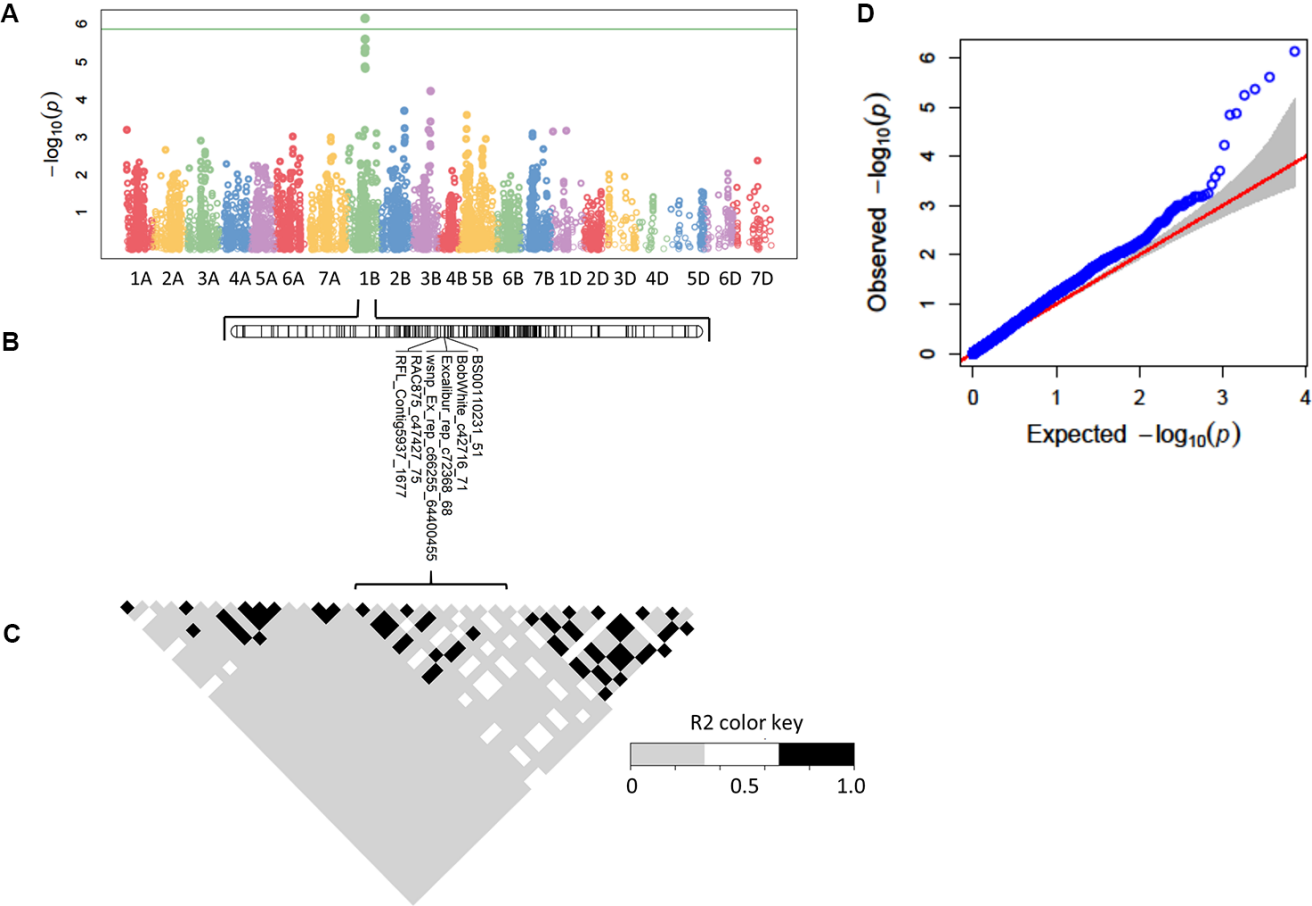
Single nucleotide polymorphism (SNP) significantly associated with STB resistance in winter wheat identified by genome-wide association study (GWAS) with GLM model. **(A)** Manhattan plot; **(B)** Linkage map of Chromosome 1B; **(C)** Linkage disequilibrium plot; **(D)** Quantile-quantile plot.

**Figure 5:**
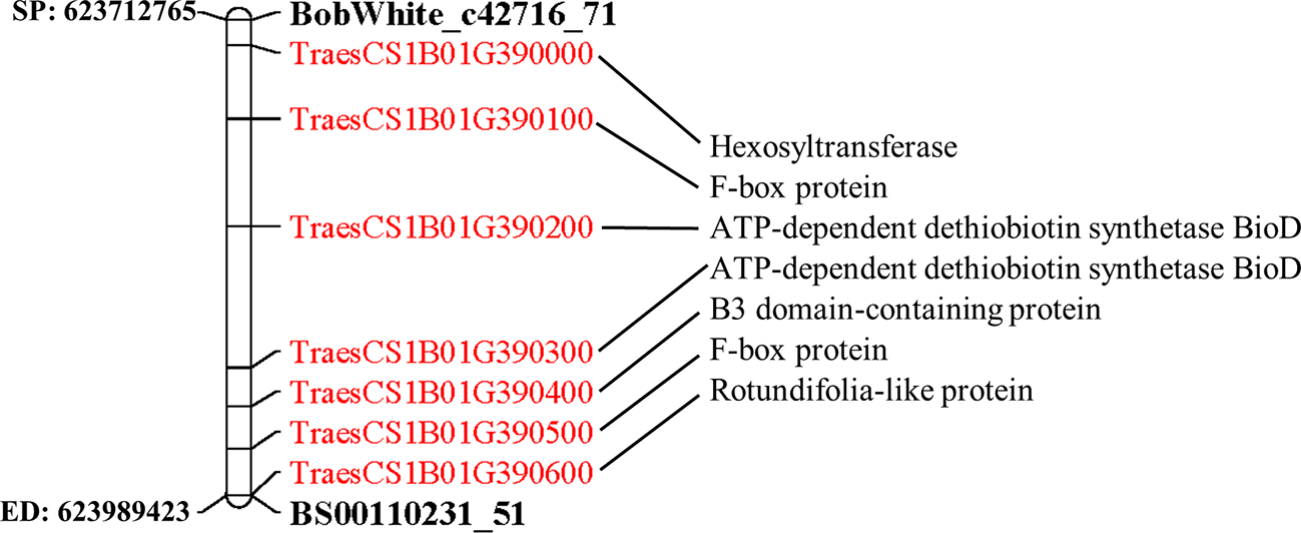
Wheat chromosome 1B representing the physical position (in bp) of the flanking markers and genes localized within these markers. SP, start position (BobWhite_c42716_71); ED end position (BS00110231).

The GWAS was performed using the GLM model, and both population structure and kinship (K) were taken into account to control pseudo associations (**Figure 4**). As is shown by the Manhattan plot and quantile-quantile plot (QQ plot) (**Figures 4A, D**), six significant (FDR < 0.05) SNP markers for rAUDPC of STB were detected on chromosome 1B. The identified QTL was verified using four additional GWAS models, namely, MLM, MLMM, FarmCPU, and Super and the QTL was found to be statistically significant (FDR < 0.05) in MLMM, FarmCPU, and Super results (**Supplementary Figure 2**). All six markers are located within a 2 cM distance on chromosome 1B (97–99 cM), thus, suggesting that it could potentially be a single QTL (**Table 1**, **Figure 5**). Additional QTL were also identified on chromosome 1A, 2B, 3A, and 5A in at least two GWAS models each (**Table 1**).

**Table 1:** Summary of the significant SNPs marker identified with different models which are associated with Septoria tritici blotch (STB) resistance in GWAS analysis with 175 winter wheat genotypes.

SNP marker name	Chr	Model	Position (cM)	MAF	Alleles	R^2^	Allelic effecvt	Physical location
BobWhite_c1361_1187	1A	FarmCPU**** Super****	13.73	0.14	A/G	–	0.16	1525253
BobWhite_c42716_71	1B	FarmCPU**** GLM*** MLM* MLMM*** Super****	97.71	0.46	A/G	0.11	0.02	623712765
wsnp_Ex_rep_c66255_64400455	1B	GLM**	97.71	0.47	A/G	0.09	−0.01	623729791
RFL_Contig5937_1677	1B	GLM**	99.07	0.45	A/G	0.08	−0.01	623730512
RAC875_c47427_75	1B	GLM*** MLM*	99.07	0.47	A/G	0.10	−0.01	623731255
Excalibur_rep_c72368_68	1B	GLM*** MLM*	97.71	0.46	T/C	0.09	−0.003	623770763
BS00110231_51	1B	GLM**	97.36	0.43	T/G	0.09	0.01	623989423
wsnp_Ex_c22423_31615798	2B	FarmCPU*** Super***	96.99	0.37	A/C	–	0.08	215593752
wsnp_Ex_c5929_10402147	3A	FarmCPU**** Super****	86.16	0.31	T/C	–	−0.09	481018206
Excalibur_c17553_84	5A	FarmCPU*** Super***	43.27	0.35	C/T	–	0.09	375375809

Chr, chromosome; MAF, minor allele frequency, physical location – start positions (in bp) of the markers on the chromosomes in the assembly IWGSC Refseq v1. FDR-adjusted p value *0.05, **0.01, ***0.001, ****0.0001. The percentage of variation (R^2^) explained by the GLM model was calculated as the difference between the R^2^ of the GAPIT model with and without the associated SNP. Allelic effect estimates the additive contribution of the tested marker and were obtained primarily from the GLM model when available else from FarmCPU model.

### Candidate Genes Located in the QTL on Chromosome 1B

In total, seven candidate genes were identified that were localized within the GWAS identified loci on chromosome 1B (**Figure 5**). Among these genes, two genes code for F-box protein (TraesCS1B01G390100, TraesCS1B01G390500) and two genes for ATP-dependent dethiobiotin synthetase BioD (TraesCS1B01G390200, TraesCS1B01G390300). The other three genes code for B3 domain-containing protein (TraesCS1B01G390400), Rotundifolia-like protein (TraesCS1B01G390600), and Hexosyltransferase (TraesCS1B01G390000).

### Genomic Prediction

GP method was applied based on all SNPs, and the prediction of the genomic breeding value for each line was evaluated using 500 randomly generated train/test sets. The average correlation between observed tolerance to STB and predicted STB by GP was 0.47 in a model with no significant markers included as fixed effects. The GWAS results were used to select markers to fit as fixed effects. Significant markers were pooled from the GLM, MLM, MLMM, FarmCPU, and Super models. The six significant SNP markers identified in proximity to each other on chromosome 1B were reduced to the marker BobWhite_c42716_71 on the basis of the lowest FDR-adjusted p-value. In total, five significant markers were used as candidates for modeling with fixed effects (**Table 1**).

All possible combinations of the five GWAS-selected SNP markers were evaluated, in subset sizes from one marker to all five used as fixed effects (**Table 2**). The highest average prediction accuracy (0.62) was obtained from a model that included all five markers as fixed effects. Among the models with reduced number of markers (1–3 markers) set as fixed effects, the models using three GWAS-selected markers performed better compared to the models using one or two markers. The prediction accuracy thus increased on average from 0.48 for one marker added as fixed effect to 0.54 for three markers. Out of the three marker models, the best performing model was a model that included the following three markers BobWhite_c1361_1187, BobWhite_c42716_71, and Excalibur_c17553_84 with a prediction accuracy of 0.59 (**Supplementary Table 2**). In comparison, the model that did not use GWAS-selected markers as fixed effects, and the models that used randomly selected markers (regardless of GWAS significance), performed on average worse than both the GWAS-assisted models and the model with all markers set as random effects (**Table 2**).

**Table 2:** Summary of rrBLUP-based GWAS-assisted genomic prediction models of STB resistance scored in 175 winter wheat genotypes.

Number of markers set as fixed effects	Type of marker selection for fixed effects			
	Markers selected by significance in GWAS		Completely random selection of markers	
	Average model accuracy	95% confidence interval of the mean	Average model accuracy	95% confidence interval of the mean
0	0.47	N/A	N/A	N/A
1	0.48	[0.44, 0.51]	0.44	[0.43, 0.44]
2	0.51	[0.49, 0.53]	0.44	[0.43, 0.45]
3	0.54	[0.52, 0.56]	0.45	[0.42, 0.48]
4	0.58	[0.55, 0.61]	0.43	[0.41, 0.45]
5	0.62	N/A	0.44	[0.41, 0.47]

The models utilized permutations of 1 to 5 markers in significant association with STB resistance identified in the same population. The models were compared against a model containing no fixed effects and a series of models that sampled equally sized subsets of random markers, where each subset of random markers was repeated five times. All models were validated against the same set of 80/20 training/test sets (N = 500). The zero and five GWAS-selected marker models were only repeated once, and thus have no confidence interval data.

### Haplotype Analysis

**Figure 6:**
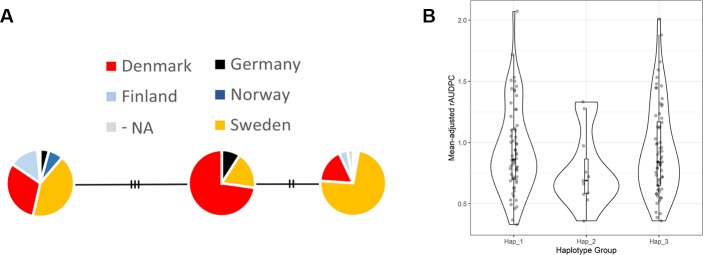
Haplotype variants identified from the QTL on chromosome 1B. **(A)** Haplotype network with nodes denoted as pie charts and **(B)** range of distribution of STB resistance of genotypes in each variant.

Haplotype analysis was performed to identify haplotype variants for the QTL identified on chromosome 1B with six significant markers. Haplotype variants were detected with the software DNAsp (Rozas et al., 2017). In total, 19 haplotype variants were detected with number of genotypes ranging from 1 to 71 in each variant. Of these, 3 haplotype variants were selected with at least five or more genotypic counts/genotypes (**Supplementary Table 1**). Thereafter, haplotype network was constructed with the TCS algorithm in the software PopART (Leigh et al., 2015) (**Figure 6**). The analysis revealed that Hap_2 had the lowest mean disease score of 0.77 compared to Hap_1 (0.96) and Hap_3 (0.95). Hap_2 had 11 genotypes of which 8 originated from Denmark, 2 from Sweden, and 1 from Germany. Most of the genotypes from Denmark had high resistance while one of the two genotypes from Sweden had high resistance.

## Discussion

STB is one of the most important winter wheat diseases in Northern Europe, and cultivars with higher levels of resistance which is stable and effective across environments are needed. Whereas individual Stb genes are not currently effective against *Z. tritici* populations in Europe (Arraiano et al., 2009), the identification of new QTL for STB resistance and incorporation of resistance into elite winter wheat cultivars is crucial. To this end, the current study analyzed 175 winter wheat genotypes of Nordic origin for STB resistance under controlled conditions at the seedling stage. Our results revealed that the NordGen genebank has a highly valuable and genetically diverse collection of germplasm comprising resistance to STB. This germplasm mainly originates from Sweden, Denmark, Finland, and Norway composing 56.2%, 25.5%, 9.6%, and 3.4% of all analyzed germplasm, respectively, and released approximately between 1900 and 2000. Population structure analysis revealed three clusters associated with geographical origin. Finish and Norwegian genotypes formed one cluster, the second cluster contains mainly Swedish genotypes while genotypes from Denmark and Germany segregated into the third cluster (**Figure 2A**). In addition, the result from the PCA data showed that the modern wheat cultivars exhibited a higher level of resistance in comparison to older released cultivars (**Figures 2B C**). This result indicated that the breeding progress for STB resistance over the last decades probably occurred by the gradual accumulation of genes with a minor effect, as is the case also in the American germplasm (Jlibene et al., 1994; Camacho-Casas et al., 1995). Similarly, the characterization of old Tunisian durum wheat accessions for resistance to STB identified resistant germplasm and four new resistant genes (Ferjaoui et al., 2015). The authors, therefore, suggested that the old Tunisian durum wheat accessions harbor novel resistance genes that can be introgressed into the modern cultivars. The results from our work highlight the potential of old germplasm as novel sources of resistance to STB for winter wheat breeding programs in Northern Europe.

A QTL associated with STB resistance identified by GWAS in this study was mapped on chromosome 1B. Previous studies have mapped *Stb11* on the short arm of chromosome 1B in *TE9111* (Chartrain et al., 2005) and remapped *Stb2* was also located close to or at Stb11 locus in Vernopolis (Liu et al., 2013). *StbWW* identified in three DH populations, was also mapped on chromosome 1BS at or near *Stb11*. Raman et al. (2009) identified eight SNPs associated with STB resistance and one was mapped on chromosome 1B in European winter wheat collection. Goudemand et al. (2013) mapped two QTL on 1B (one 1BS and one 1BL) chromosome in bi-parental crosses. Recently, Vagndorf et al. (2017) identified QTL QStb.*NS-1B* located on the long arm of chromosome 1B by GWAS of Danish cultivars and breeding lines that were characterized over three years in three locations in Denmark for STB. In this study, one QTL was mapped on the long arm of chromosome 1B which is in close physical proximity to the QTL QStb.*NS-1B*. Thus, it can be postulated that it is the same QTL as identified previously. However, our study identified this QTL for quantitative resistance at the seedling stage under controlled conditions while the study by Vagndorf et al. (2017) identified the same QTL in field trials for adult plant resistance.

The other QTL associated with STB resistance identified in this study were located on chromosomes 1A, 2B, 3A, and 5A. QTL 1A, 2B, and 5A were mapped on the short arm of the respective chromosomes and QTL on 3A was mapped on the long arm. Goudemand et al. (2013) mapped two Meta-QTL (MQTL1 and MQTL6) on chromosomes 1A and 2B and another QTL (QTL8) on chromosome 5A for STB resistance which were in close physical proximity to the QTL mapped (1A, 2B, and 5A) in this study. The MQTL1 was associated with STB resistance both in adult and seedling stages whereas QTL8 was only associated with adult and MQTL6 was only associated with seedling stage resistance.

The QTL on chromosome 3A in our study was found in close physical proximity to the previously reported QTL (*QStb.risø-3A.2*) which was associated with STB resistance both in adult and seedling stages (Brown et al., 2015). Thus, our study further confirms the role of the identified QTL at the seedling stage. Introgression of these QTL in winter wheat cultivars will provide both seedling and adult plant stage resistance to STB.

In the present work, we identified seven candidate genes with putative roles in resistance to STB in wheat (**Figure 5**). Two of the identified genes (*TraesCS1B01G390100* and *TraesCS1B01G390500*) were associated with F-box proteins which plays a key role in plant immune responses through the involvement in hormone pathways (Yu et al., 2007). Two F-box proteins, *COI1* (Xie et al., 1998) and *SON1* (Kim and Delaney, 2002), have been demonstrated to have a role in plant defense in Arabidopsis plants. In our previous work, we identified candidate genes associated with STB resistance by integrating QTL mapping and transcriptome profiling, wherein, the F- box proteins were among the most represented in all identified QTL regions (Odilbekov et al., 2019). The other two genes identified in this work were related to ATP-dependent dethiobiotin synthetase BioD (*TraesCS1B01G390200* and *TraesCS1B01G390300*). ATP-dependent dethiobiotin synthetase BioD is involved in the first step of the sub-pathway that synthesizes biotin from 7,8-diaminononanoate. Li et al. (2012) demonstrated that biotin deficiency results in light-dependent spontaneous cell death and modulates defense gene expression in Arabidopsis plants. The other putative genes identified in the present work were B3 domain-containing protein (*TraesCS1B01G390400*). The B3 domain has been found in several transcription factors specific to higher plant species (Waltner et al., 2005). Wang et al. (2015) found that the B3 domain of BPH29 gene was associated with insect brown planthopper resistance in rice. Also, they have shown that during the infestation, the *RBPH54* triggers the salicylic acid signaling pathway and suppresses the jasmonic acid pathway, which is similar to biotrophic pathogens.

In the previous studies, prediction accuracy of GS models was found to be improved for example by increasing the training population size, testing the models on test populations genetically closely related to the training population, implementing a different GS algorithm, increasing the marker density or combining significantly associated markers as fixed effects (Solberg et al., 2008; Norman et al., 2018). In this work, we evaluated the prediction accuracy of GP models when GWAS markers were included as fixed effects. When GWAS markers obtained from different GWAS models were included as fixed effects, the accuracy of GP was significantly improved (**Table 2**). The results also suggest that including two or more GWAS markers as fixed effects significantly increases the accuracy of the GP models. Our results corroborate the trends in accuracy improvements seen in the previous studies integrating GWAS and GP in winter wheat (Herter et al., 2019) maize (Bian and Holland, 2017), and rice (Spindel et al., 2016).

Finally, this and the previous studies (Daetwyler et al., 2014; Crossa et al., 2016) have shown that GP can be used to obtain GEBVs for economically important traits in landraces by training models on a subset of landraces that are phenotyped. There are several hundred thousand landraces stored in genebanks worldwide, and thus, advanced methods, such as GP will enable high-throughput evaluation of landraces to identify those with superior resistance traits. The identified landraces can then be included in the wheat breeding programs to perform GP-based progeny selection.

## Conclusions

This study leads to the conclusion that the wheat genotypes stored at NordGen are a genetically diverse resource. The highly resistant genotypes serve as potential donors for improving commercial cultivars in the Nordic and Baltic Sea Region countries. The significant SNP markers can be used for marker-assisted selection of STB resistance at the seedling stage in wheat breeding. The genes identified by GWAS approach can serve as candidate genes for improving STB resistance in wheat through functional studies. In addition, the results indicate that integrating GWAS with GP could facilitate further improvement of GP accuracy thereby improving the selection efficiency of the breeding program.

## Data Availability Statement

The genotypic data can be assessed from the following link https://doi.org/10.6084/m9.figshare.10184468.

## Author Contributions

AC conceived and planned the study and performed GWAS, haplotype and GP analysis. FO and RA performed germplasm characterization. AK performed GWAS-assisted GP analysis. JS selected genotypes and performed genotyping. FO performed statistical analysis and wrote the first draft. All authors contributed in the interpretation of the data and in writing the manuscript.

## Funding

This project was funded by Lantmännen Research Foundation (2016F010), Einar Nilssons Stiftelse, and SLU Grogrund.

## Conflict of Interest

The authors declare that the research was conducted in the absence of any commercial or financial relationships that could be construed as a potential conflict of interest.
