# Supplementary material for: GWAS-Assisted Genomic Prediction to Predict Resistance to Septoria Tritici Blotch in Nordic Winter Wheat at Seedling Stage
Source: Front Genet. 2019 Nov 26;10:1224. doi: 10.3389/fgene.2019.01224 (PMC6901976; doi:10.3389/fgene.2019.01224)

Supplementary Figure 1. Response of the differential wheat genotypes with known Stb genes to Swedish isolate of *Zymoseptoria tritici.* The differential set was described previously (Ghaffary et al., 2018)

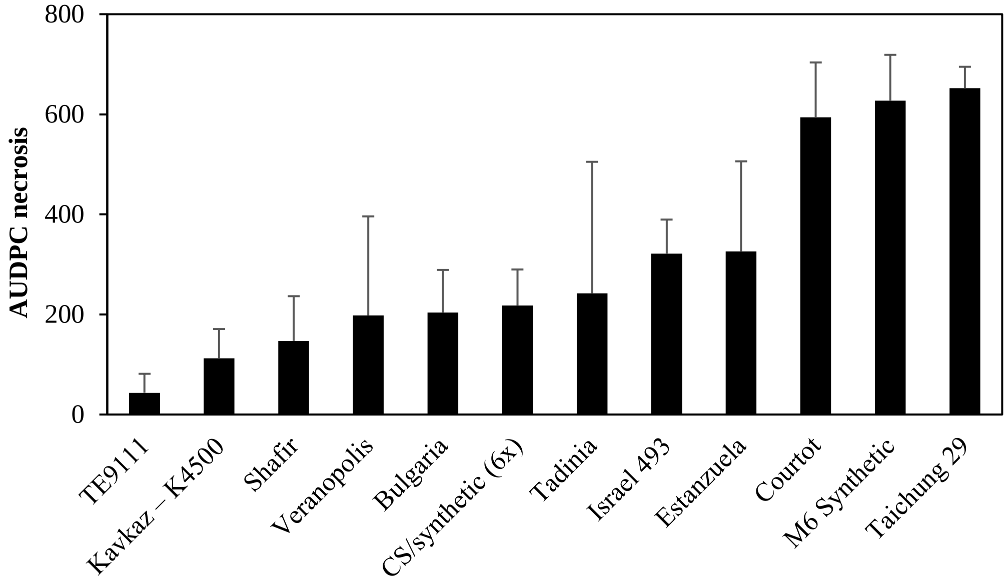

Ghaffary, S.M.T., Chawade, A., and Singh, P.K. (2018). Practical breeding strategies to improve resistance to Septoria tritici blotch of wheat. *Euphytica* 214(7). doi: 10.1007/s10681-018-2205-4.

Supplementary Figure 2. Single nucleotide polymorphism (SNP) significantly associated with STB resistance in winter wheat identified by genome-wide association study (GWAS) with four different models. (A) MLM; (B) FarmCPU; (C) MLMM; (D) Super.

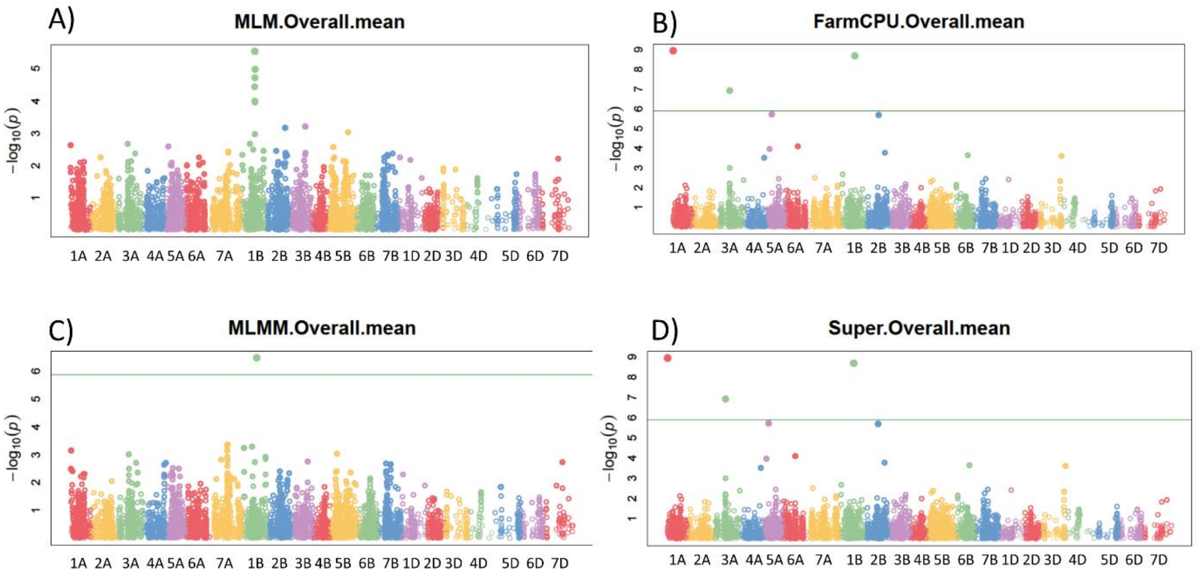

Supplement: Supplementary file 1 [file Presentation_1.pdf]
